# Berberine promotes immunological outcomes and decreases neuroinflammation in the experimental model of multiple sclerosis through the expansion of Treg and Th2 cells

**DOI:** 10.1002/iid3.766

**Published:** 2023-01-13

**Authors:** Maryam J. Tavaf, Azita Soltanmohammadi, Simin Zargarani, Esmaeil Yazdanpanah, Bizhan Sadighimoghaddam, Bahman Yousefi, Hamid R. Sameni, Dariush Haghmorad

**Affiliations:** ^1^ Department of Immunology, School of Medicine Semnan University of Medical Sciences Semnan Iran; ^2^ Department of Immunology and Allergy, Immunology Research Center Mashhad University of Medical Sciences Mashhad Iran; ^3^ Department of Tissue Engineering and Applied Cellular Sciences, School of Medicine Semnan University of Medical Sciences Semnan Iran; ^4^ Cancer Research Center Semnan University of Medical Sciences Semnan Iran

**Keywords:** Berberine, experimental autoimmune encephalomyelitis, multiple sclerosis, myelin oligodendrocyte glycoprotein

## Abstract

**Introduction:**

Among the most frequent demyelinating autoimmune disorders of the central nervous system (CNS) is multiple sclerosis. Experimental autoimmune encephalomyelitis (EAE) is used as an animal model of multiple sclerosis. Berberine is an alkaloid found in some medicinal plants with anti‐inflammatory effects.

**Methods:**

C57BL/6 female mice were used and divided into three groups: (1) The control group received PBS, (2) the low‐dose treatment group received 10 mg/kg of berberine, and (3) The high‐dose treatment group received 30 mg/kg of berberine. Myelin Oligodendrocyte Glycoprotein and complete Freund's adjuvant were subcutaneously administered to induce EAE. Mice were given intraperitoneal injections of pertussis toxin on the day of immunization and 2 days later. Histological studies showed low lymphocyte infiltration and demyelination of CNS in the treated groups.

**Results:**

The clinical scores of the treatment group with low‐dose berberine (T1: 2 ± 0.13) and high‐dose berberine (T2: 1.5 ± 0.14) were significantly (*p* < .001) lower than the control group (CTRL: 4.5 ± 0.13). Treatment groups decreased pro‐inflammatory cytokines (IFN‐γ, TNF‐α, interleukin [IL]‐17) (*p* < .001) as well as increased anti‐inflammatory cytokine expression (IL‐4, IL‐10, IL‐27, IL‐33, IL‐35, TGF‐β) (*p* < .01) when compared to the CTRL group. Treatment groups with berberine reduced expression of the Th1 and Th17 cytokines and transcription factors (*p* < .001) and increased expression of transcription factors and Th2 and Treg cytokines (*p* < .01) in contrast to CTRL group.

**Conclusion:**

Berberine appears to have a protective effect on disease development and alleviating disease status in EAE, which appears to be due to the cell expansion and function of Treg and Th2 cells in addition to berberine's anti‐inflammatory properties.

## Graphical abstract

In this manuscript, we assessed the beneficial effects of berberine treatment with low and high doses on amelioration of experimental autoimmune encephalomyelitis. We made use of a mouse experimental autoimmune encephalomyelitis (EAE) induced model with the administration of berberine. The results indicate that berberine ameliorates the EAE condition by affecting Th2/Treg functions and proliferation as well as Th1/Th17 cells.

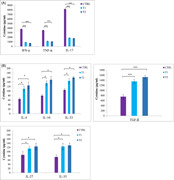

## 1 INTRODUCTION

Multiple sclerosis is an immune‐mediated disorder of the central nervous system (CNS), determined by demyelination as a result of blood–brain barrier (BBB) dysfunction and invasion of mononuclear cells into the white matter.^1^ The immunopathogenesis of Multiple sclerosis remains to be elucidated^2^ but evidence supports that the interaction of environmental and genetic influences, in addition the etiology, are of utmost importance for the development of MS.^3^ The pathologic hallmark of MS is associated with demyelinated plaques within the CNS, expression of inflammatory mediators, gliosis, and neurodegeneration in all stages of multiple sclerosis.^2^, ^4^ A common model of MS is experimental autoimmune encephalomyelitis (EAE), a T‐cell‐mediated model of autoimmune demyelination of the CNS to test various therapeutically and restorative approaches.^5^ Immunization with CNS tissue or myelin peptides emulsified in the adjuvant can cause active EAE as well as passive EAE induced by myelin‐specific CD4^+^ T cells generated in a multitude of species and strains.^6^

Accumulating evidence indicates that adaptive immunity especially Th1/Th17‐mediated inflammation plays an important part in the development of MS and a lot of other autoimmune diseases such as Rheumatoid arthritis, Systemic lupus erythematosus, and type I diabetes.^7^ Cytokines are important elements of the immunological inflammatory response and deregulated cytokine responses are an undeniable factor in driving CNS inflammation in MS patients.^8^ Th2 cells, characterized by the expression of the transcription factor GATA3, which predominantly produces IL‐4/IL‐10, originally were considered to exert a suppressive role in the pathogenesis of autoimmune demyelination.^9^ Additionally, Treg characterized by expression of the transcriptional factor FoxP3 and production of TGF‐β and IL‐10, has been considered pivotal for controlling autoinflammatory diseases during MS and EAE.^10^

IL‐27, IL‐33, and IL‐35 are newly discovered anti‐inflammatory/immunosuppressive cytokines that both IL‐27 and IL‐35 belong to anti‐inflammatory cytokines of the IL‐12 while IL‐33 is a new member of the IL‐1 superfamily of cytokines.^11^ IL‐27 typically produced by antigen‐presenting cells (APCs) can decrease EAE severity through a variety of processes, such as diminishing Th17 cell differentiation and enhancing the production of IL‐10.^12^, ^13^ In other anti‐inflammatory cytokines, IL‐33 treatment may have a positive effect on the EAE treatment process by switching from predominantly Th1/Th17 pathogenic cells to Th2 and Treg cells.^14^ IL‐35 is another suppressor cytokine mainly released by Treg cells that are required for the maximum inhibitory effect of mouse Treg cells in vitro and in vivo.^15^

To date, a variety of anti‐inflammatory and immunoregulatory therapies are used to prevent the progress of MS, but they are only several partially effective treatments to modify the disease course. Therefore, it is necessary to develop a novel safe, and effective approach to manage the disorder, emphasis on preventing axonal degeneration and neuronal loss.^16^ Berberine, an important protoberberine isoquinoline alkaloid, is originally derived from many herb therapies such as *Hydrastis Canadensis* with a long history of numerous years of utilization in traditional therapies.^17^ Berberine, both in its pure shape and as a major component of therapeutic plants, as demonstrated to exhibit a broad spectrum of pharmacological and biological properties such as antibiotic activity, antiplatelet aggregation properties, antidiarrheal, antiarrhythmia, antihypertension, antitumor.^18^, ^19^, ^20^ In addition, berberine has been shown to exert immune‐modulatory and neuroprotective activities in inflammatory and autoimmune disorders.^17^, ^21^ Established and emerging data showed that berberine treatment can ameliorate clinical and pathological parameters of EAE via decreasing Th1/Th17 cytokine production and Th1 and Th17 cell development^22^ and reducing the permeability of BBB,^23^ suggesting the potential role of berberine in the treatment of neurodegenerative diseases.

To our knowledge, this was the first research to clarify the effect of berberine on the immune responses of Th2/Treg cells, and in this study, we try to investigate the effect of berberine on the secretion of cytokines such as IL‐4, IL‐10, IL‐27, IL‐33, IL‐35, TGF‐β and related transcription factors including STAT6, GATA3, FoxP3, and PD1.

In this study, we evaluate the potential effects of berberine in the treatment of the experimental mouse model of MS, moreover, assessment of immunological mechanisms involved in this disease.

## 2 MATERIALS AND METHODS

### 2.1 Mice

Wild‐type 8 to 10‐week‐old C57BL/6 mice were obtained from the Royan Institute for Biotechnology (Isfahan, Iran). All mice were housed in a particular pathogen‐free facility at a temperature of 23 ± 2°C, a relative humidity of 50 ± 5%, and a 12‐h light/dark cycle under standard circumstances, with standard mouse food and water available at all times. All experiments were carried out in agreement with protocols certified by the ethics committee for animal experimentation at Semnan University of Medical Sciences (IR.SEMUMS.REC.1396.191).

### 2.2 Induction, treatment, and evaluation of EAE

Mice received a subcutaneous injection of 250 µg of myelin oligodendrocyte glycoprotein (MOG_35–55_) (BioBasic, Canada) emulsified in complete Freund's adjuvant (Sigma‐Aldrich) containing 4 mg/ml Mycobacterium tuberculosis H37Ra (Difco Laboratories) at the base of the tail. Then 250 ng pertussis toxins (Sigma‐Aldrich) were administered i.p. on the day of immunization and repeated 2 days afterward. Berberine purchased from Sigma‐Aldrich (Sigma‐Aldrich, and SKU Number: 14050) and the doses of berberine chose from previous study.^23^, ^24^ Twenty‐four mice were randomly separated into three groups: Control (*n* = 8), Low dose treatment group (T1, 10 mg/kg berberine, *n* = 8), and high‐dose treatment group (T2, 30 mg/kg berberine, *n* = 8). Berberine treatment by oral gavage was initiated from the first day of disease induction until the last day. Clinical indications of EAE were observed and the weight of mice was evaluated was assessed daily up to 25 days after immunization. Mice were scored for clinical assessment with the following scale: 0, no symptoms; 1 =  incomplete failure of tail tonicity; 2 = complete failure of tail tonicity; 3 = flaccid tail and unusual gait; 4 = hind limb paralysis; 5 = hind limb paralysis with hind body paresis; 6 = hind and foreleg paralysis; 7 = moribund or death.^25^, ^26^

### 2.3 Histological studies

The mice were anesthetized on Day 25 following the induction of EAE with ketamine (150 mg/kg) and xylazine (10 mg/kg), then their spleen and lymph nodes were removed and PBS containing 4% paraformaldehyde was used to perform an intracardiac perfusion. After being embedded in paraffin, brains were removed and stained with Luxol Fast Blue (LFB) and Hematoxylin & Eosin (H&E) to assess demyelination and leukocyte infiltration, respectively. After that, the sections underwent a blinded light microscopy evaluation.

The following scale was used to assess inflammation; 0, no inflammatory cells; 1, a few numbered inflammatory cells; 2, inflammatory infiltrates' organizational structure surrounding blood vessels; and 3, large perivascular cuffing that extends into the nearby parenchyma, or parenchymal infiltration with lacking a clear cuffing. The brain demyelination was graded in the manner previously described; 0, normal white matter; 1, distinct foci; 2, a few demyelination locales; and 3, extensive areas of demyelination.^27^

### 2.4 Cell culture and BrdU proliferation assay

On the basis of the assessment of BrdU incorporation during DNA synthesis, T cell proliferation was also assessed using a colorimetric immunoassay. On Day 25 following immunization, spleens and peripheral lymph nodes (inguinal and axillary) were isolated from C57BL/6 mice. Ammonium chloride was used to lyse red blood cells. Cell suspensions were obtained using 10% fetal bovine serum (FBS), 100 U/ml penicillin, and 100 mg/ml streptomycin in RPMI 1640 medium (all reagents purchased from Sigma). Cell suspensions were cultured in medium alone or with MOG_35–55_ (20 μg/ml) on round‐bottomed 24‐well and 96‐well plates at 37°C for 72 h.

After incubation, proliferation was assayed with the Cell Proliferation ELISA (BrdU) Kit (Roche Applied Science) according to the manufacturer's recommendations. The intensity of the color was measured within 5 min utilizing an ELISA plate reader, at 450 nm.

### 2.5 ELISA for cytokine detection

Supernatants from cultured cells were collected after 72 h, and cytokine concentrations (IL‐4, IL‐10, IL‐27, IL‐33, IL‐35, TGF‐β, IFN‐γ, IL‐17, and TNF‐α) were measured by ELISA to the manufacturer's recommendations (eBioscience). Briefly, capture antibody was diluted in coating buffer, coated in plate's wells, and incubated for an overnight duration at 4°C. Plates were washed and blocked with ELISA diluent for 1 h at room temperature. Following a 2 h incubation period at room temperature, samples and standards were incubated with a biotinylated secondary antibody for 1 h.

Then plates were incubated with Avidin‐HRP for 30 min and by using tetramethylbenzidine (TMB) plates were developed. Finally, the reaction with the stop solution was stopped and plates were measured at 450 nm using the microplate reader (Stat Fax 2100 Awareness). Based on measurements of multiple recombinant cytokine concentrations, standard curves were developed.

### 2.6 Quantitative real‐time PCR

To determine the expression of cytokines and transcription factors of infiltrated immune cells, the brain was removed on Day 25 after EAE induction from diseased mice. The brains of each mouse were pulverized individually in phosphate buffer while the cells were isolated using nylon mesh. For RNA extraction, the solution was centrifuged at 3000 g for 10 min. The cell pellet that was obtained was then suspended in TriPure Isolation Reagent (Roche Applied Science). cDNA synthesis was carried out by PrimeScript™ RT reagent Kit (Takara Bio Inc.) according to the manufacturer's instructions. As well as RNA extraction and cDNA synthesis of mononuclear cells obtained from spleen and lymph nodes was performed.

Real‐time PCR was carried out using the SYBR Green qPCR Master Mix (Ampliqon) with suitable primers (Table 1) synthesized by Metabion Company. Table 1 presents the sequences of the produced primers. Reactions were performed by Applied Biosystems™ StepOnePlus™ (Thermo Fisher Scientific) to find the relative quantity of mRNA expression in accordance to the β2 microglobulin reference gene.

**Table 1:** Sequences of primer which used in study

Genes	Forward	Reverse
IFN‐γ	GTCATTGAAAGCCTAGAAAGTC	TGCCAGTTCCTCCAGATA
STAT4	ATTCTGACTTTGGACTTG	TTCTAATTGTTGGACTTGA
T‐bet	GTTCAACCAGCACCAGAC	ACGGTGAAGGACAGGAAT
IL‐17	ACTACCTCAACCGTTCCA	GCTTCCCAGATCACAGAG
STAT3	CACCTTGGATTGAGAGTC	AGGAATCGGCTATATTGC
ROR‐γt	CACACCTCACAAATTGAAG	GATAACCCCGTAGTGGA
TNF‐α	CTGTCTATACCAACAGAC	TGTTCATAGCATCATCGT
IL‐4	ATGCACGGAGATGGATGT	ACCTTGGAAGCCCTACAG
STAT6	TTGGTAGTGCCCTCTGAG	GAGACATGATCTGGGATATACA
GATA3	CCTGTGGGCTGTACTACAAG	CGGTTTCGGGTCTGGATG
IL‐10	AGCAGGTGAAGAGTGATT	GCAGTTGATGAAGATGTCA
IL‐33	AGTACAGCATTCAAGACC	TGGAGTTGGAATACTTCATT
TGF‐β	CGCAACAACGCCATCTAT	TGCTTCCCGAATGTCTGA
FoxP3	AAGTGGCAGAGAGGTATT	CAGAGTCAGGAGAAGTTG
PD1	GTAACAGAGAGAATCCTG	CATGATACCAATGACCAT
B2m	TATCCAGAAAACCCCTCAAA	CGTAGCAGTTCAGTATGTTC

Relative quantification delta‐delta CT calculation are shown as fold changes vs the control group.

### 2.7 Statistical analysis

Comparison of the treatment groups versus control mice on the occurrence of clinical symptoms was carried out via two‐way repeated‐measures analysis of variance (ANOVA). One‐way ANOVA followed by Tukey multiple comparison tests was performed for analysis between groups. The normality of the data was confirmed by the Shapiro–Wilk test. The data were analyzed using SPSS 26. Data were displayed as mean ± SEM. The definition for statistical significance were *p < .05, **p < .01, ***p < .001.

## 3 RESULTS

### 3.1 Berberine treatment ameliorates the clinical severity of the disease

**Figure 1:**
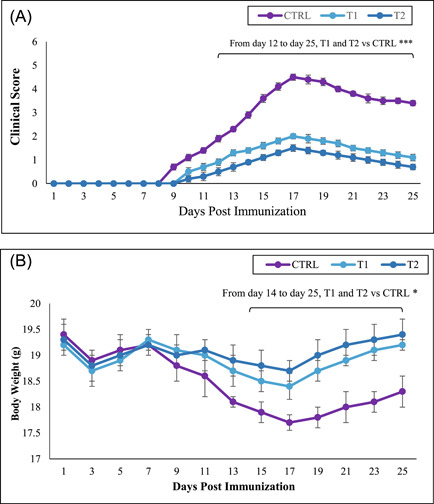
Berberine inhibited the development of experimental autoimmune encephalomyelitis (EAE) in MOG‐immunized C57BL/6 mice. Female C57BL/6 mice were treated with 10 and 30 mg/kg berberine in treatment groups on Day 1 simultaneous with EAE induction until Day 25 postimmunization. Mice were monitored for signs of EAE, and the results for all mice, were presented as (A) mean clinical score and (B) body weight. Results were expressed as mean ± SEM. *p < .05; ***p < .001, compared with control group. 24 mice were divided into three groups: (1) Control group (CTRL), (2) low‐dose berberine treatment group (T1), and (3) high‐dose berberine treatment group (T2).

MOG‐immunized C57BL/6 female mice aged 8–10 weeks were randomly divided into three groups. These groups were assigned as control group, low‐dose treatment group (T1), and high‐dose treatment group (T2, 30 mg/kg berberine). All mice were observed for indications of EAE and a remarkable reduction in disease severity. The results for all of the groups, showed as mean clinical score and body weight in Figure 1.

Berberine in low and high doses was capable of reducing the disease's severity. The treatment groups were able to significantly decrease the severity of disability and paralysis compared to the control group. Surprisingly, high‐dose berberine‐treated mice were able to greatly reduce the symptoms of the disease.

The clinical scores (on Day 17, maximal score) of the treatment group with low dose berberine (T1: 2 ± 0.13) and high dose berberine (T2: 1.5 ± 0.14) were significantly (*p* < .001) lower than the control group (CTRL: 4.5 ± 0.13) (Figure 1A and Table 2). Additionally, the treatment groups effectively prevented EAE mice from losing weight. The mean body weight of the T1 and T2 groups on Day 17 (maximal score) were 18.4 ± 0.25 and 18.7 ± 0.2, respectively (*p* < .05) compared to the CTRL group with 17.7 ± 0.15 (Figure 1B). As expected, the EAE mice without any treatment exhibited clinical symptoms that ranked at the maximum score on Day 17 post‐EAE induction. In contrast, EAE mice receiving berberine (T1 and T2) showed milder symptoms of the disease.

**Table 2:** Clinical features of experimental autoimmune encephalomyelitis (EAE) in the administration of berberine

Group	Day of onset	Maximal score (score at peak)	Mean score (last day)	Cumulative disease index (CDI)
CTRL^a^	9.1 ± 0.2	4.5 ± 0.13	3.4 ± 0.12	53.1 ± 0.63
T1^b^	10.7 ± 0.3*	2 ± 0.11***	1.1 ± 0.11***	22.2 ± 0.48***
T2^c^	11.1 ± 0.4*	1.5 ± 0.13***	0.7 ± 0.1***	19.1 ± 0.51***

*Note*: Data were expressed as mean ± SEM. All experiment groups compared with CTRL group. *p < .05; **p < .01; ***p < .001.

^a^ CTRL: Control group EAE induced received soybean oil.

^b^ T1: Low dose Berberine treatment group.

^c^ T2: High dose Berberine treatment group.

### 3.2 Berberine administration reduces CNS inflammation and demyelination

**Figure 2:**
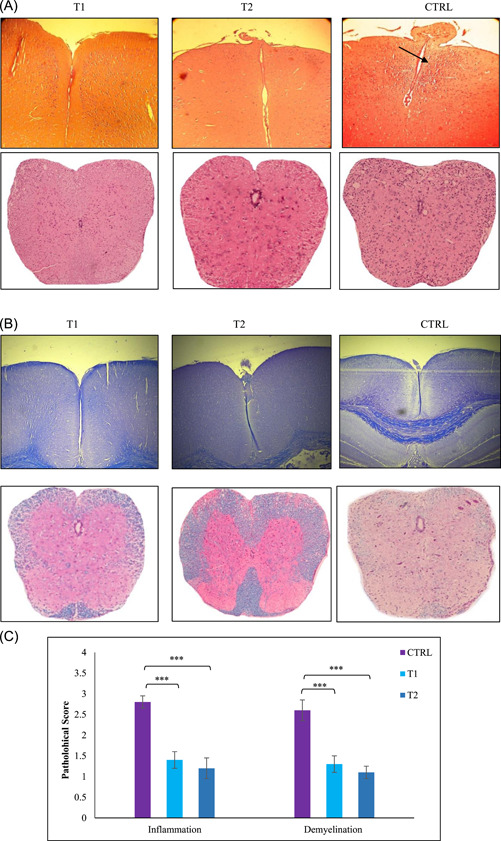
Comparative histopathology of the brain demonstrated berberine suppresses central nervous system (CNS) inflammation and demyelination. Histopathological evaluation of the brain from treated groups (low and high dose Berberine) and control was performed. Brains from each group, were collected on Day 25 postimmunization, fixed in paraformaldehyde, and embedded in paraffin. Five‐micrometer sections from different regions of the brain from each of the groups were stained with (A) hematoxylin & eosin (H&E) to enumerate infiltrating leukocytes and with (B) Luxol fast blue to assess demyelination by Light microscope (10X, scale bar). (C) CNS inflammatory foci and infiltrating inflammatory cells were quantified. Pathological scores including inflammation and demyelination were analyzed and shown with a bar graph as mean scores of pathological inflammation or demyelination ± SEM. Data are representative of three independent experiments. ***p < .001 compared with control group. 24 mice were divided into three groups: (1) Control group (CTRL), (2) low‐dose berberine treatment group (T1), and (3), high‐dose berberine treatment group (T2).

The leukocyte infiltration and demyelination were evaluated by H&E and Luxol fast blue. H&E staining revealed a significant reduction in the frequency of CNS infiltrating leukocytes in treatment groups compared with the control group (1.4 ± 0.2, 1.2 ± 0.25, and 2.8 ± 0.15, respectively; *p* < .001) (Figure 2C). As well, Luxol fast blue staining showed the scores for demyelinating in the control and treatment groups as 2.6 ± 0.25, 1.3 ± 0.2, and 1.3 ± 0.15, respectively; *p* < .001 (Figure 2C). The results, point out that demyelination of the brain in treatment groups markedly reduced regarding the control group. The leukocyte infiltration and demyelination in the brain of treated mice and control groups are shown in Figure 2A,B. The results indicated more inflammatory infiltration in the control group in comparison with treatment groups. In addition, significantly attenuated demyelination was observed in treated groups versus large plaque of demyelination in the control group.

### 3.3 Berberine treatment decreased T‐cell proliferation

**Figure 3:**
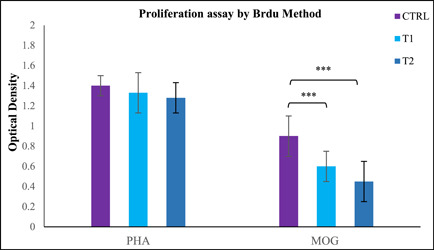
Berberine suppresses T‐cell proliferation. Spleen cells were harvested on day 25 postimmunization and cultured in PHA 20 μg/ml) as a positive control or with myelin oligodendrocyte glycoprotein (MOG) (20 μg/ml) for 72 h on 96‐well plates. Proliferation responses were tested using a cell proliferation ELISA, BrdU (colorimetric) kit (Roche Applied Science). Proliferation assay was conducted in triplicate wells. Data presented as mean optical density ± SEM. ***p < .001 compared with control group. 24 mice were divided into three groups: (1) Control group (CTRL), (2) low‐dose Berberine treatment group (T1), and (3) high‐dose berberine treatment group (T2).

The cell proliferation assay of T‐cells extracted from the spleen was done by a Cell Proliferation ELISA, BrdU (colorimetric) kit. Cultivation of samples was carried out with MOG (20 μg/ml) and PHA (20 μg/ml) as a positive control. Cell proliferation of all sample groups with PHA was almost close. The results displayed that mononuclear cells of berberine‐treated mice had the significantly reduced capacity for proliferation in the spleen and lymph nodes when compared to the CTRL group (Figure 3).

### 3.4 Berberine downregulates the expression of pro‐inflammatory cytokines and upregulates anti‐inflammatory cytokines production

**Figure 4:**
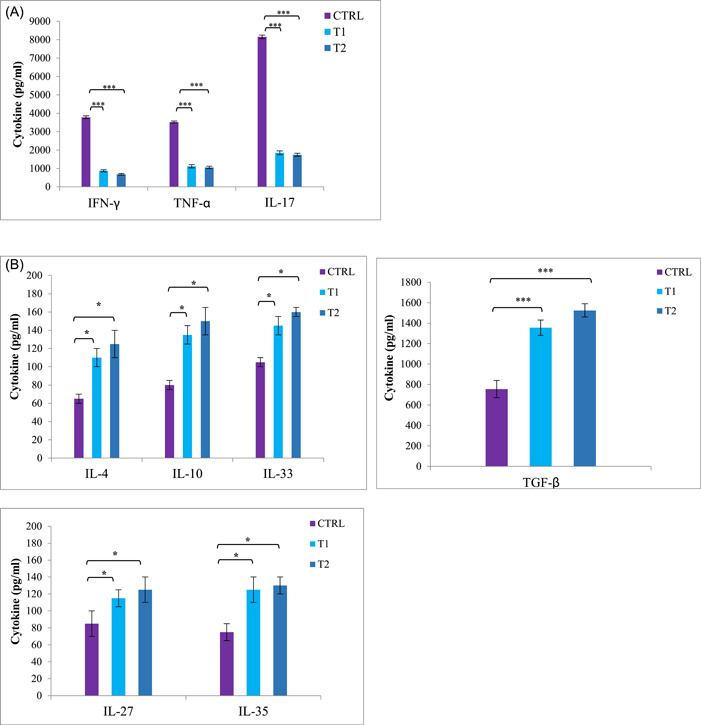
Berberine suppressed pro‐inflammatory cytokines production and enhanced anti‐inflammatory cytokines production in splenocytes and lymph nodes from experimental autoimmune encephalomyelitis (EAE) mice. Splenocytes and lymph nodes from immunized mice from all groups (24 mice) were isolated on Day 25 postimmunization and restimulated with myelin oligodendrocyte glycoprotein (MOG)_35–55_ (20 μg/ml) for 72 h. Culture supernatants were collected and indicated cytokine levels were measured by ELISA. Cytokine assays were conducted in duplicate wells. (A) Pro‐inflammatory cytokines as IFN‐γ TNF‐α, and IL‐17 ± SEM and (B) anti‐inflammatory cytokines as IL‐4, IL‐10, IL‐27, IL‐33, IL‐35, and TGF‐β were measured from supernatants of cultures from splenocytes and lymph nodes. Results from lymph nodes were similar to splenocytes and data was not shown. *p < .05; ***p < .001 compared with control group. 24 mice were divided into three groups: (1) Control group (CTRL), (2) low‐dose berberine treatment group (T1), and (3) high‐dose berberine treatment group (T2).

Cytokine levels of cultured supernatants were measured using ELISA. The results indicated that the treatment groups decreased pro‐inflammatory cytokines (IFN‐γ, TNF‐α, and IL‐17) as well as increased anti‐inflammatory cytokine expression (IL‐4, IL‐10, IL‐27, IL‐33, IL‐35, and TGF‐β) when compared to the CTRL group (Figure 4). These data indicate that treatment with low and high doses of berberine (T1 and T2 groups) significantly reduces the cytokines production of Th1 and Th17 and also increases the cytokines production of Th2 and Treg. As a result, the balance of pro‐inflammatory and anti‐inflammatory cytokines in the EAE model changes during berberine treatment.

### 3.5 Berberine increased expression of Treg and Th2 cytokines and transcription factors

**Figure 5:**
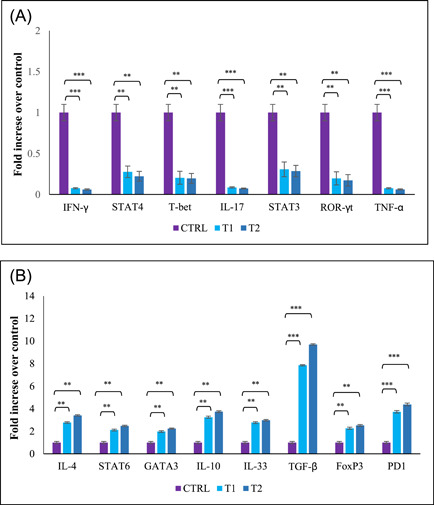
Gene expression of cytokines and transcription factors in central nervous system (CNS). On Day 25 postimmunization, brains, and spinal cords were collected and mRNA levels of cytokines and transcription factors were assessed by real‐time quantitative PCR. The assay was run in triplicate and fold change expression of genes was determined compared control group. (A) Th1 and Th17 related cytokines and transcription factors; IFN‐γ, STAT4, T‐bet, IL‐17, STAT3, ROR‐γt, TNF‐α (B) Th2 and Treg related cytokines and transcription factors; IL‐4, STAT6, GATA3, IL‐10, IL‐33, TGF‐β, FoxP3, and PD1. Results were expressed as fold change compared with the control group. ** *p* < .01; ***p < .001. 24 mice were divided into three groups: (1) Control group (CTRL), (2) low‐dose berberine treatment group (T1), and (3) high‐dose berberine treatment group (T2).

Quantitative real‐time PCR was used to evaluate the expression of brain and spinal cord mRNA expression of cytokines and transcription factors secreted by T cells infiltrating the CNS. The data offered that, treatment groups with berberine reduced expression of the Th1 and Th17 cytokines and transcription factors (IFN‐γ, STAT4, T‐bet, IL‐17, STAT3, ROR‐γt, and TNF‐α) and increased expression of transcription factors and Th2 and Treg cytokines (IL‐4, STAT6, GATA3, IL‐10, IL‐33, TGF‐β, FoxP3, and PD1) in contrast to CTRL group (Figure 5). This alteration in cytokines and transcription factors established cytokine ELISA results obtained from lymphoid organs, including the spleen and lymph nodes.

## 4 DISCUSSION

In recent years, berberine has been utilized in traditional medicine to treat a variety of infectious disorders. Due to the anti‐inflammatory and antioxidant effects of berberine, this drug has been used in the treatment of the animal model of MS.^23^

This study assessed the efficacy of berberine in the management of EAE. No effective treatment for MS has been found so far, and finding a cure is still challenging.^28^ EAE is an animal model of MS that has been developed to evaluate different therapy strategies.^29^ To date, a variety of anti‐inflammatory and immunoregulatory therapies are used to prevent the progress of MS, but they are only several partially effective treatments to modify the disease course. Therefore, it is necessary to develop a novel safe, and effective approach to improve the disease concentrating on preventing neuronal loss and axonal degradation.^16^ Among different treatment approaches, targeted drug therapy pointed out impressing advancement; although, the efficacy of these approaches regarding side effects is still a major parameter limiting them. A suitable alternate strategy for this objective is nutritional intervention by the administration of bioactive food components to patients. There are several significant dietary flavonoids that are known to have immune‐modulating properties. This flavonoids feature suggests that they may 1 day be used to prevent and/or treat autoimmune disorders.^30^

In the previous studies, the results supported that berberine has made neuroprotective effects in an animal model of MS. These results, are presented by enhanced neurophysiological function, and less severe demyelination and inflammatory cell infiltration in the spinal cord. These findings are demonstrating the protective effects of berberine.^28^

In a natural immune system, there is an accurate balance between effector T cells with various functions. More specifically, the balance between inflammatory factors and tolerance plays an important role in the development of protective immune responses to pathogens without interfering with immune tolerance to self‐antigens. Loss of this balance is an essential criterion responsible for the development of autoimmune disorders. Furthermore, investigating novel approaches that focus on these influential factors should provide great therapeutic potential for treating autoimmune disorders.^30^ Adaptive immunity especially Th1/Th17‐mediated inflammation play a crucial role in the progression of MS and a lot of other autoimmune diseases such as Rheumatoid arthritis, Systemic lupus erythematosus and type I diabetes.^7^ Some cytokines are critical components of the immune inflammatory process and impaired production of these cytokines triggers inflammation in the CNS.^31^

Recently, berberine is effective in the regulation of the differentiation of Th1 and Th17 cells and improved the severity of EAE.^32^, ^33^ Additionally, berberine can decrease the clinical severity of EAE by reducing BBB permeability, inflammatory cells infiltration into CNS and as a result reducing CNS inflammation.^16^ Ma et al. demonstrated that administration of oral berberine at the beginning of the disease is effective in improving the severity of EAE in C57BL/6 mice.^23^ In our study, the neurological symptoms and clinical scores of the treatment groups (T1 and T2) were significantly lower than the control group. Also, the infiltration of immune cells and demyelination on the brain of EAE induced mice were decreased in treatment groups. These results confirmed that treatment with berberine efficiently improved the disease in the animal model of MS.

In this study, we showed that berberine can suppress the pro‐inflammatory cytokine derived from Th1 and Th17 such as IFN‐γ, IL‐17, and TNF‐α. In this study, by evaluating of the gene expression involved in the Th1 and Th17 signaling pathways, including STAT4, T‐bet, STAT3, and ROR‐γt, we found that the expression of these genes was reduced in berberine‐treated mice. Th1 and Th17 cells are strongly involved in the pathogenesis of MS and its animal model, EAE.^34^, ^35^ Moreover, berberine can enhance the anti‐inflammatory cytokine and induced the expression of genes related to the Treg and Th2 cells including IL‐4, STAT6, GATA3, IL‐10, IL‐33, TGF‐β, FoxP3, and PD1. The results of this study also revealed that berberine could cause expanded production of anti‐inflammatory cytokines in Treg and Th2 that ameliorate EAE severity symptoms in treated groups. The role of Treg cells in MS was first documented by studies in the EAE because the adoptive transfer of Treg cells improves disease progression, while depletion of these cells worsened the disease.^36^ Functional disorders of Treg cells producing IL‐10 and TGF‐β are involved in the development of MS and EAE.^37^

T cell activation results in differentiation into the Th17 or Treg phenotype under the effect of a particular cytokine environment.^38^ Determining the pathogenic roles of Th17 cells and the modulatory actions of Treg cells has also made substantial progress in recent years.^39^ These discoveries have paved the way for the development of novel treatment strategies that target particular Th17/Treg balance mediators and assist in resetting that balance to improve immunological homeostasis and limit the development of autoimmune disease.^40^, ^41^

Scientific results suggest that anti‐inflammatory drugs by affecting a single target couldn't play a role in alleviating autoimmune conditions. In contrast, combinations of traditional Chinese medicine that target the immune/inflammatory pathway may be more effective in ideally regulating the defective immune system. In addition, the anti‐inflammatory properties of berberine elicited by EAE studies may provide a new way to use it as a starting point for initiating pathway‐based immunomodulation therapies.

In conclusion, these findings imply that berberine plays an important role in holding the balance between pro‐inflammatory and anti‐inflammatory T cells, which confirms previous studies. The immune system has a variety of mechanisms involved in controlling the development and differentiation of activated T cells in processes such as anergy, death, and regulation. Indeed, in the current research, we found direct evidence that berberine could reduce the expression of inflammatory cytokines in Th1 and Th17 cells, meanwhile increasing the activity of Treg and Th2 cells and enhancing secretion of regulatory cytokines from these cells. In this research, we discovered berberine regulates the functions of effective subsets of CD4^+^ T cells by targeting transcription factors and their transducers. These new findings make it easier to comprehend of the underlying mechanisms of berberine in reducing T cell‐mediated autoimmune disorders and suggested useful applications of berberine in autoimmune diseases therapy, which offers an efficient strategy to both prevention and treatment of autoimmune diseases.

## AUTHOR CONTRIBUTIONS

Dariush Haghmorad conceived and developed the experiments. Maryam J. Tavaf, Azita Soltanmohammadi, Simin Zargarani, Bizhan Sadighimoghaddam, Bahman Yousefi, and Hamid Reza Sameni carried out the experiments. Maryam J. Tavaf, Esmaeil Yazdanpanah, and Dariush Haghmorad performed the evaluation and analyzed the experimental data. Maryam J. Tavaf and Esmaeil Yazdanpanah wrote the article. Dariush Haghmorad revised the writing.

## CONFLICT OF INTEREST

The authors declare no conflict of interest.

## References

[1] Kamma E , Lasisi W , Libner C , Ng HS , Plemel JR . Central nervous system macrophages in progressive multiple sclerosis: relationship to neurodegeneration and therapeutics. J Neuroinflam. 2022;19(1):45. 10.1186/s12974-022-02408-y PMC8830034 35144628

[2] Yadav SK , Mindur JE , Ito K , Dhib‐Jalbut S . Advances in the immunopathogenesis of multiple sclerosis. Curr Opin Neurol. 2015;28(3):206‐219. 10.1097/wco.0000000000000205 25887768

[3] Høglund RA . Multiple sclerosis and the role of immune cells. World J Exp Med. 2014;4(3):27‐37. 10.5493/wjem.v4.i3.27 25254187 PMC4172701

[4] Psenicka MW , Smith BC , Tinkey RA , Williams JL . Connecting neuroinflammation and neurodegeneration in multiple sclerosis: are oligodendrocyte precursor cells a nexus of disease? Front Cell Neurosci. 2021;15:654284. 10.3389/fncel.2021.654284 34234647 PMC8255483

[5] Titus HE , Chen Y , Podojil JR , et al. Pre‐clinical and clinical implications of “Inside‐Out” vs. “Outside‐In” paradigms in multiple sclerosis etiopathogenesis. Front Cell Neurosci. 2020;14:599717. 10.3389/fncel.2020.599717 33192332 PMC7654287

[6] Denic A , Johnson AJ , Bieber AJ , Warrington AE , Rodriguez M , Pirko I . The relevance of animal models in multiple sclerosis research. Pathophysiology. 2011;18(1):21‐29. 10.1016/j.pathophys.2010.04.004 20537877 PMC3858209

[7] Li C , Xi Y , Li S , et al. Berberine ameliorates TNBS induced colitis by inhibiting inflammatory responses and Th1/Th17 differentiation. Mol Immunol. 2015;67(2 Pt B):444‐454. 10.1016/j.molimm.2015.07.013 26224047

[8] Matejuk A , Vandenbark AA , Offner H . Cross‐talk of the CNS with immune cells and functions in health and disease. Front Neurol. 2021;12:672455. 10.3389/fneur.2021.672455 34135852 PMC8200536

[9] Benallegue N , Kebir H , Alvarez JI . Neuroinflammation: extinguishing a blaze of T cells. Immunol Rev. 2022;311:151‐176. 10.1111/imr.13122 35909230 PMC9489683

[10] Fletcher JM , Lalor SJ , Sweeney CM , Tubridy N , Mills KHG . T cells in multiple sclerosis and experimental autoimmune encephalomyelitis. Clin Exp Immunol. 2010;162(1):1‐11. 10.1111/j.1365-2249.2010.04143.x 20682002 PMC2990924

[11] Kourko O , Seaver K , Odoardi N , Basta S , Gee K . IL‐27, IL‐30, and IL‐35: a cytokine triumvirate in cancer. Front Oncol. 2019;9:969. 10.3389/fonc.2019.00969 31681561 PMC6797860

[12] Hirahara K , Ghoreschi K , Yang X‐P , et al. Interleukin‐27 priming of T cells controls IL‐17 production in trans via induction of the ligand PD‐L1. Immunity. 2012;36(6):1017‐1030. 10.1016/j.immuni.2012.03.024 22726954 PMC3785111

[13] Legroux L , Arbour N . Multiple sclerosis and T lymphocytes: an entangled story. J Neuroimmune Pharmacol. 2015;10(4):528‐546. 10.1007/s11481-015-9614-0 25946987 PMC5052065

[14] Rao X , Hua F , Zhang L , et al. Dual roles of interleukin‐33 in cognitive function by regulating central nervous system inflammation. J Transl Med. 2022;20(1):369. 10.1186/s12967-022-03570-w 35974336 PMC9382782

[15] Collison LW , Chaturvedi V , Henderson AL , et al. IL‐35‐mediated induction of a potent regulatory T cell population. Nature Immunol. 2010;11(12):1093‐1101. 10.1038/ni.1952 20953201 PMC3008395

[16] Jiang Y , Wu A , Zhu C , et al. The protective effect of berberine against neuronal damage by inhibiting matrix metalloproteinase‐9 and laminin degradation in experimental autoimmune encephalomyelitis. Neurol Res. 2013;35(4):360‐368. 10.1179/1743132812y.0000000156 23540404

[17] Zhang M , Feng L , Li J , Chen L . Therapeutic potential and mechanisms of berberine in cardiovascular disease. Current Pharmacology Reports. 2016;2(6):281‐292. 10.1007/s40495-016-0070-1

[18] Wang X , Jiang S , Sun Q . Effects of berberine on human rheumatoid arthritis fibroblast‐like synoviocytes. Exp Biol Med. 2011;236(7):859‐866. 10.1258/ebm.2011.010366 21676922

[19] Habtemariam S . Berberine and inflammatory bowel disease: a concise review. Pharmacol Res. 2016;113(Pt A):592‐599. 10.1016/j.phrs.2016.09.041 27697643

[20] Behl T , Singh S , Sharma N , et al. Expatiating the pharmacological and nanotechnological aspects of the alkaloidal drug berberine: current and future trends. Molecules. 2022;27(12):3705. 10.3390/molecules27123705 35744831 PMC9229453

[21] Och A , Podgórski R , Nowak R . Biological activity of Berberine‐A summary update. Toxins. 2020;12(11):713. 10.3390/toxins12110713 33198257 PMC7697704

[22] Yan F , Wang L , Shi Y , et al. Berberine promotes recovery of colitis and inhibits inflammatory responses in colonic macrophages and epithelial cells in DSS‐treated mice. Am J Physiol Gastrointest Liver Physiol. 2012;302(5):G504‐G514. 10.1152/ajpgi.00312.2011 22173918 PMC3311435

[23] Ma X , Jiang Y , Wu A , et al. Berberine attenuates experimental autoimmune encephalomyelitis in C57 BL/6 mice. PLoS One. 2010;5(10):e13489. 10.1371/journal.pone.0013489 20976070 PMC2957444

[24] Chandirasegaran G , Elanchezhiyan C , Ghosh K , Sethupathy S . Berberine chloride ameliorates oxidative stress, inflammation and apoptosis in the pancreas of Streptozotocin induced diabetic rats. Biomed Pharmacother Biomed Pharmacother. 2017;95:175‐185. 10.1016/j.biopha.2017.08.040 28843149

[25] Mahmoudi MB , Mahmoudi M , Rab SZT , et al. Calcium intervention ameliorates experimental model of multiple sclerosis. Oman Med J. 2014;29(3):185‐189. 10.5001/omj.2014.46 24936267 PMC4052390

[26] Haghmorad D , Yazdanpanah E , Sadighimoghaddam B , et al. Kombucha ameliorates experimental autoimmune encephalomyelitis through activation of Treg and Th2 cells. Acta Neurol Belg. 2021;121(6):1685‐1692. 10.1007/s13760-020-01475-3 32812134

[27] Haghmorad D , Yazdanpanah E , Jadid Tavaf M , et al. Prevention and treatment of experimental autoimmune encephalomyelitis induced mice with 1, 25‐dihydroxyvitamin D3. Neurol Res. 2019;41:943‐957. 10.1080/01616412.2019.1650218 31402771

[28] Luo J , Chen R , Zeng S , et al. The effects of berberine on a murine model of multiple sclerosis and the SPHK1/S1P signaling pathway. Biochem Biophys Res Commun. 2017;490(3):927‐932. 28655617 10.1016/j.bbrc.2017.06.142

[29] Constantinescu CS , Farooqi N , O'Brien K , Gran B . Experimental autoimmune encephalomyelitis (EAE) as a model for multiple sclerosis (MS): EAE as model for MS. Br J Pharmacol. 2011;164(4):1079‐1106. 21371012 10.1111/j.1476-5381.2011.01302.x PMC3229753

[30] Wang J , Niu X , Wu C , Wu D . Naringenin modifies the development of lineage‐specific effector CD4+ T cells. Front Immunol. 2018;9:2267. 30327657 10.3389/fimmu.2018.02267 PMC6174281

[31] Trenova A , Slavov G . Cytokines in multiple sclerosis–possible targets for immune therapies. J Neurol Exp Neurosci. 2016;1(2):25‐29.

[32] Qin X , Guo BT , Wan B , et al. Regulation of Th1 and Th17 cell differentiation and amelioration of experimental autoimmune encephalomyelitis by natural product compound berberine. J Immunol. 2010;185(3):1855‐1863. 20622114 10.4049/jimmunol.0903853

[33] Ehteshamfar SM , Akhbari M , Afshari JT , et al. Anti‐inflammatory and immune‐modulatory impacts of berberine on activation of autoreactive T cells in autoimmune inflammation. J Cell Mol Med. 2020;24(23):13573‐13588. 10.1111/jcmm.16049 33135395 PMC7754052

[34] Loos J , Schmaul S , Noll TM , et al. Functional characteristics of Th1, Th17, and ex‐Th17 cells in EAE revealed by intravital two‐photon microscopy. J Neuroinflammation. 2020;17(1):357. 10.1186/s12974-020-02021-x 33243290 PMC7694901

[35] Wagner CA , Roqué PJ , Goverman JM . Pathogenic T cell cytokines in multiple sclerosis. J Exp Med. 2020;217(1):e20190460. 10.1084/jem.20190460 31611252 PMC7037255

[36] Ghorbani MM , Farazmandfar T , Abediankenari S , Hassannia H , Maleki Z , Shahbazi M . Treatment of EAE mice with Treg, G‐MDSC and IL‐2: a new insight into cell therapy for multiple sclerosis. Immunotherapy. 2022;14(10):789‐798. 10.2217/imt-2021-0045 35678041

[37] Kleinewietfeld M , Hafler DA . Regulatory T cells in autoimmune neuroinflammation. Immunol Rev. 2014;259(1):231‐244. 10.1111/imr.12169 24712469 PMC3990868

[38] Wang C , Collins M , Kuchroo VK . Effector T cell differentiation: are master regulators of effector T cells still the masters? Curr Opin Immunol. 2015;37:6‐10. 10.1016/j.coi.2015.08.001 26319196

[39] Josefowicz SZ , Lu LF , Rudensky AY . Regulatory T cells: mechanisms of differentiation and function. Annu Rev Immunol. 2012;30:531‐564. 10.1146/annurev.immunol.25.022106.141623 22224781 PMC6066374

[40] Fasching P , Stradner M , Graninger W , Dejaco C , Fessler J . Therapeutic potential of targeting the Th17/Treg axis in autoimmune disorders. Molecules. 2017;22(1):134. 10.3390/molecules22010134 28098832 PMC6155880

[41] Goswami TK , Singh M , Dhawan M , et al. Regulatory T cells (Tregs) and their therapeutic potential against autoimmune disorders—advances and challenges. Hum Vaccines Immunother. 2022;18(1):2035117. 10.1080/21645515.2022.2035117 PMC9009914 35240914

